# Multifunctional Tannic Acid/Silver Nanoparticle-Based Mucoadhesive Hydrogel for Improved Local Treatment of HSV Infection: In Vitro and In Vivo Studies

**DOI:** 10.3390/ijms19020387

**Published:** 2018-01-28

**Authors:** Emilia Szymańska, Piotr Orłowski, Katarzyna Winnicka, Emilia Tomaszewska, Piotr Bąska, Grzegorz Celichowski, Jarosław Grobelny, Anna Basa, Małgorzata Krzyżowska

**Affiliations:** 1 Department of Pharmaceutical Technology, Medical University of Białystok, Mickiewicza 2c, 15-222 Białystok, Poland; kwin@umb.edu.pl; 2 Military Institute of Hygiene and Epidemiology, Kozielska 4, 01-163 Warsaw, Poland; porlowski@wihe.waw.pl; 3 Department of Materials Technology and Chemistry, Faculty of Chemistry, University of Łódź, Pomorska 163, 90-236 Łódź, Poland; etomaszewska@uni.lodz.pl (E.T.); gcelichowski@uni.lodz.pl (G.C.); jgrobel@uni.lodz.pl (J.G.); 4 Department of Preclinical Sciences, Faculty of Veterinary Medicine, Warsaw University of Life Sciences, Ciszewskiego 8, 02-786 Warsaw, Poland; piotr_baska@sggw.pl; 5 Institute of Chemistry, University of Białystok, Ciołkowskiego 1K, 15-245 Białystok, Poland; abasa@uwb.edu.pl; 6 Wrocław Research Centre EIT+, Stablowicka 147, 54-066 Wrocław, Poland

**Keywords:** tannic acid modified silver nanoparticles, HSV 1/2, antiherpes activity, hydrogel, mucoadhesiveness, Carbopol 974P

## Abstract

Mucoadhesive gelling systems with tannic acid modified silver nanoparticles were developed for effective treatment of herpes virus infections. To increase nanoparticle residence time after local application, semi solid formulations designed from generally regarded as safe (GRAS) excipients were investigated for their rheological and mechanical properties followed with ex vivo mucoadhesive behavior to the porcine vaginal mucosa. Particular effort was made to evaluate the activity of nanoparticle-based hydrogels toward herpes simplex virus (HSV) type 1 and 2 infection in vitro in immortal human keratinocyte cell line and in vivo using murine model of HSV-2 genital infection. The effect of infectivity was determined by real time quantitative polymerase chain reaction, plaque assay, inactivation, attachment, penetration and cell-to-cell assessments. All analyzed nanoparticle-based hydrogels exhibited pseudoplastic and thixotropic properties. Viscosity and mechanical measurements of hydrogels were found to correlate with the mucoadhesive properties. The results confirmed the ability of nanoparticle-based hydrogels to affect viral attachment, impede penetration and cell-to-cell transmission, although profound differences in the activity evoked by tested preparations toward HSV-1 and HSV-2 were noted. In addition, these findings demonstrated the in vivo potential of tannic acid modified silver nanoparticle-based hydrogels for vaginal treatment of HSV-2 genital infection.

## 1. Introduction

Herpes simplex virus (HSV), classified as type 1 and 2, is highly prevalent contagious pathogen affecting skin and mucosal tissues of up to 67% of adolescents and adults worldwide [1,2]. HSV-1 infection, which is predominantly spread by oral-to-oral contacts, usually occurs during childhood causing Herpes labialis (with symptoms recognized as “cold sores”), although it may be associated with more serious complications, including ocular herpes or encephalitis [3,4]. Contrarily, HSV-2 is practically entirely sexually transmitted and is therefore most closely related to genital herpes characterized by the presence of painful ulcerative or vesicular lesions [5]. HSV-2 infections, which are more common in women than in men [5,6], were found to be closely associated with elevated risk of acquiring and transmitting HIV infection [7]. Both types of HSV persist in a latent state within the cell nucleus with the risk of periodical reactivation especially in patients with weakened immune system [8]. The potential hazard of HSV transmission is increased considerably among people with asymptomatic shedding.

In recent years, prevalence of HSV infections has gradually increased and antiviral resistance on conventional antiviral drugs (e.g., acyclovir) has been reported, particularly among patients exposed to a long term therapy [2,7]. Hence, effort should be made to develop novel therapeutic approaches in anti-herpes treatment. Currently, the most convenient and preferable method of HSV symptomatic treatment appears to cure lesions directly by applying local formulations with antiviral agent. Nonetheless, alternative strategies aiming at preventing either new infection or frequent recurrence of HSV episodes are simultaneously being made, including vaccines and microbicides [9,10,11].

Nanotechnology is an advanced approach to produce nanoscale drug carriers with feasible potential for uniform delivery to mucosal tissue [12,13,14]. Previously published by our group data demonstrated that silver nanoparticles modified with tannic acid (TA-AgNPs) effectively reduced HSV-2 infectivity at the vaginal mucosal surface, suggesting a potential for nanoparticles as microbicide in HSV prevention [15]. The underlying mode of TA-AgNPs action is related to an inhibition of virus attachment, penetration and cell-to-cell spreading. TA-AgNPs were additionally found to exert anti-inflammatory effect against HSV-induced inflammatory mediators [15]. Nevertheless, the fact that TA-AgNPs are produced in liquid state is a substantial drawback limiting their local application. To facilitate their application and to improve their residence time at the site of infection, using viscous semi-solid carrier is beneficial. Among semi-solid formulations, hydrogels—three-dimensional, crosslinked polymer networks—offer a great number of advantages including ease of administration, minimal influence on body functions and lubricating properties [16]. In addition, with respect to vaginal administration, hydrogels prepared from mucoadhesive polymers may provide more intimate contact between a drug carrier and the mucosal tissue, and, consequently, improve the action of active agent.

The aim of this study was to prepare and evaluate topical mucoadhesive hydrogels containing TA-AgNPs by applying Carbopol 974P, biocompatible crosslinked polyacrylic acid derivative with Generally Regarded as Safe (GRAS) category [17], as mucoadhesive gelling excipient. Precise attempt was made to assess the mucoadhesive properties of TA-AgNPs-based hydrogels measured ex vivo using porcine vaginal mucosa. In addition, this work focused on the precise examination of the effect of novel semi-solid vehicles with TA-AgNPs on inhibition of HSV-1 and HSV-2 in both in vitro and in vivo models.

## 2. Results and Discussion

### 2.1. Characterization of TA-AgNPs-Based Hydrogels

Based on the preceding cytotoxicity studies, TA-AgNPs sized 33 nm characterized by high safety profile [18] were chosen for preformulation studies of drug carriers for local delivery. It was previously demonstrated that silver nanoparticles stabilized with tannic acid displayed antiviral activity and potential as microbicide for prevention and treatment of HSV-2 infection [15,19]. Modification with tannic acid not only improved the antiviral effectiveness of silver nanoparticles in a dose-dependent manner but was also found to assign favorable anti-inflammatory properties [18]. To prolong TA-AgNPs residence time at the site of HSV infection and to improve their therapeutic antiviral efficacy, carbomer hydrogels were applied as semi-solid platforms for local delivery of nanoparticles. Carbomer polymers, crosslinked polyacrylic acid derivatives generally regarded as nontoxic and nonirritant materials [17], are extensively used in topical preparations. Among different types of carbomers, Carbopol 974P, owing to its biocompatibility and favorable mucoadhesive properties, has gained great attendance in pharmaceutical technology of local preparations [20,21]. Furthermore, Carbopol 974P is regarded to be useful in vaginal delivery of antiviral agents [22,23]. Preliminary experiments were accomplished to determine suitable amount of Carbopol 974P, polymer to TA-AgNPs ratio and optimal technological parameters. The characteristics of formulated hydrogels were summarized in Table 1.

**Figure 1:**
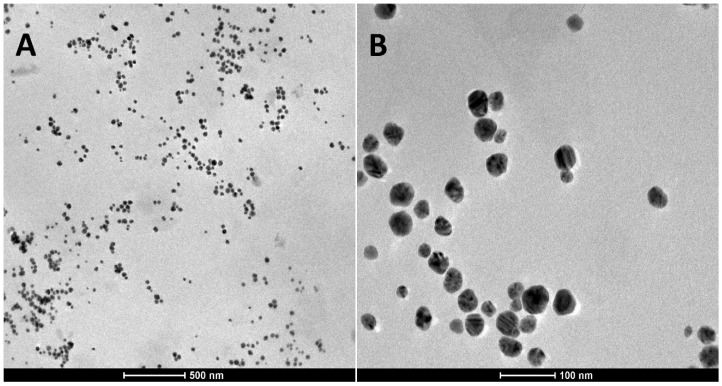
Representative TEM micrographs of hydrogel H2/NP25 with 25 ppm TA-AgNPs concentration. The scale bar is: 500 nm (**A**); and 100 nm (**B**).

Homogeneous, odorless hydrogels with no visible nanoparticles agglomeration were prepared when TA-AgNPs colloid was combined with Carbopol 974P base (with concentration range 0.4–1.0% weight/weight (*w*/*w*)) in the ratio of 1:3 (formulations H1–H4/NP25) or 1:1 (formulations H5–H8/NP50). The above-mentioned ratios were noticed to avoid alterations in nanoparticle characteristics (charge or size) and to preserve Carbopol 974P gel structure. Specifically, preliminary two-fold dilution of TA-AgNPs colloid with water to formulate H1–H4/NP25 in a ratio 1:1 with polymer base caused nanoparticle aggregation upon 48 h storage. Contrarily, higher amount of TA-AgNPs colloid within polymer network (ratio 2:1) resulted in irreversible loss of hydrogels structure, leaching TA-AgNPs out of the hydrogel matrix followed with simultaneous precipitation of nanoparticles (data not presented). Prepared formulations displayed pH values in the range from 6.3 to 7.6, prerequisite to maintain proper gel structure [24] and unlikely to induce local irritation [16]. The representative transmission electron microscopy (TEM) micrographs recorded at different magnification displayed that spherical-like nanoparticles were fairly uniformly dispersed within the Carbopol 974P matrix and minor fraction of aggregates were formed in gel network (Figure 1). The high crosslink density of Carbopol 974P network was possible stabilizing factor preventing stronger aggregation of nanoparticles. The size of the nanoparticles was in the range from 13 to 54 nm as calculated from the micrograph.

**Figure 2:**
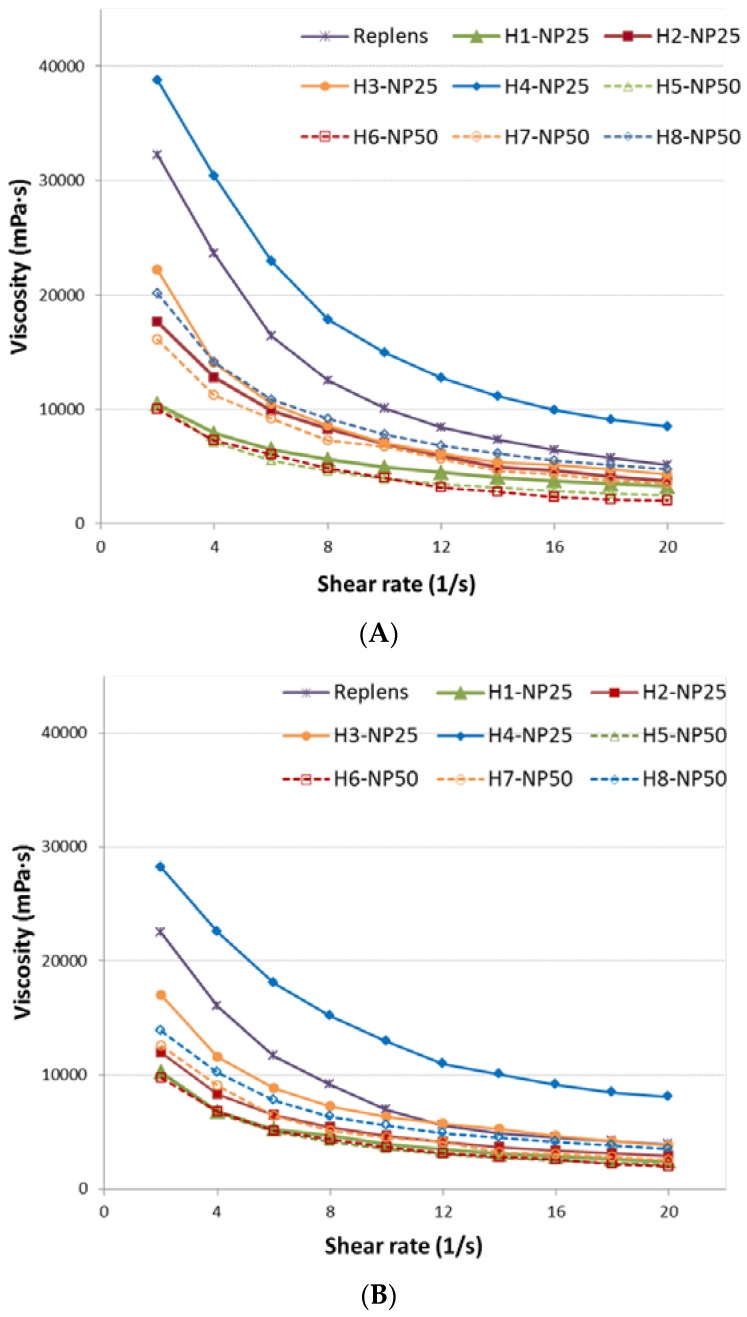
Plots of viscosity vs. shear rate of hydrogels with 25 ppm (H1/NP25–H4/NP25) or 50 ppm (H5/NP50–H8/NP50) TA-AgNPs concentration and commercially available vaginal gel Replens™ measured at: 25 °C (**A**); and 37 °C (**B**). Values are expressed as mean ± S.D (*n* = 3).

**Figure 3:**
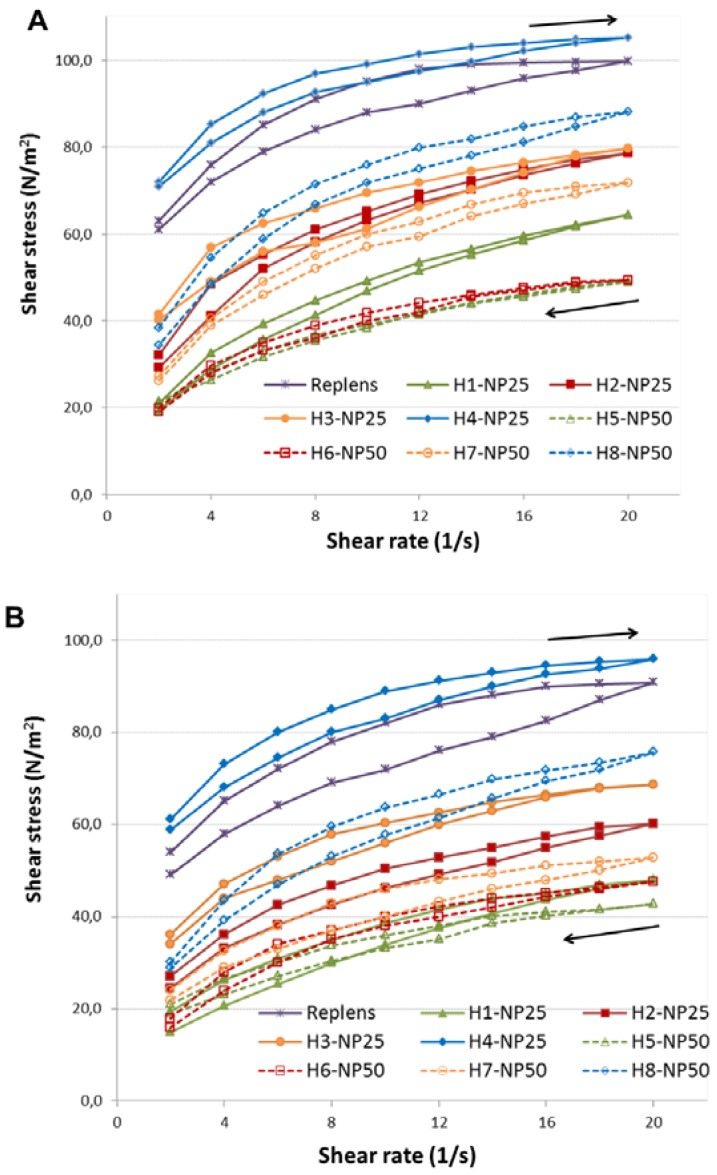
Hysteresis loops (expressed as shear stress vs. shear rate curves) of Carbopol 974P hydrogels with 25 ppm (H1–H4/NP25) or 50 ppm Ta-AgNPs concentration (H5–H8/NP50) and commercially available vaginal gel Replens™ measured at: 25 ± 1 °C (**A**); and 37 ± 1 °C (**B**). Values are expressed as mean ± S.D. (*n* = 3). Arrows indicate direction of the hysteresis loop.

The graphs of viscosity versus shear rate of the hydrogels measured at 25 ± 1 °C and 37 ± 1 °C are displayed in Figure 2, whereas the graphs of shear stress versus shear rate forming hysteresis loops are shown in Figure 3. All hydrogels presented a decrease in viscosity values with an increase in shear rate indicating non-Newtonian pseudoplastic characteristic which may facilitate product’s application. The rheological behavior of prepared hydrogels was affected by the concentration of Carbopol 974P and concentration of TA-AgNPs. Upon increasing the polymer concentration, a gradual rise in viscosity values between formulations H1–H4 and H5–H8 (with 25 ppm or 50 ppm of TA-AgNPs, respectively) was observed (Figure 2).

To determine the thixotropic behavior of hydrogels, their structural breakdown on the increasing shear rate was assessed. As displayed in Figure 3, all formulations exhibited thixotropic properties, as evidenced by characteristic shifts of the lower curves in comparison to the upper ones which refers to ability to reverse viscosity loss and gel to sol transition. In general, the rheological characteristic was found to be comparable between the samples investigated at ambient and body temperature, although substantially lower viscosities of TA-AgNPs-based hydrogels followed with their slower structural recovery (demonstrated on the graph as displacement of downward to upward curve) at 37 ± 1 °C (*p* < 0.05) were noticed (Figure 3B). The observed facility of semi-solid formulations to recover more gradually after removing the shear stress followed with loss in their strength at body temperature might favor the uniform preparations’ spreadability over the mucosal surface after local, especially vaginal, application [25].

To provide deeper insight into the internal structure of hydrogels, the structure analysis of formulations H1–H8 was assessed using a texture analyzer TA.XT. Plus. The results of hardness, adhesiveness and consistency measurements are presented in Table 1. Hardness is defined as the ability to remove the preparation from the container. Low value of hardness is associated with the ease of product removal and application, however it may reduce the retention time at the administration site [22,26]. Additionally, together with the consistency parameter—representing the work required to deform formulation during the compression of probe, hardness reflects the degree of difficulty in loss of initial hydrogel’s structure [27]. In turn, adhesiveness determines the work required to break probe-sample contact and may be an indicator of the retention time at the application site [27].

As expected, an increase in Carbopol 974P concentration gradually improved hardness and consistency values of all investigated hydrogels (Table 1). The presence of TA-AgNPs was found to impact texture properties of hydrogels. As compared to the control (0.5% (*w*/*w*) pure Carbopol 974P base), formulations H2/NP25 and H8/NP50 retained approximately 83–87% and 81–92% of their initial mechanical properties. A correlation between examined mechanical behavior and hydrogels’ viscosity was clearly displayed. Preparation H4/NP25, with the highest viscosity, was noted to exhibit the thickest and firmest structure along with the greatest adhesion ability.

All investigated hydrogels retained their rheological properties upon 14-day storage at ambient temperature in closed containers, as no substantial alterations in viscosity values followed, and no signs of precipitation were observed (data not shown).

**Figure 4:**
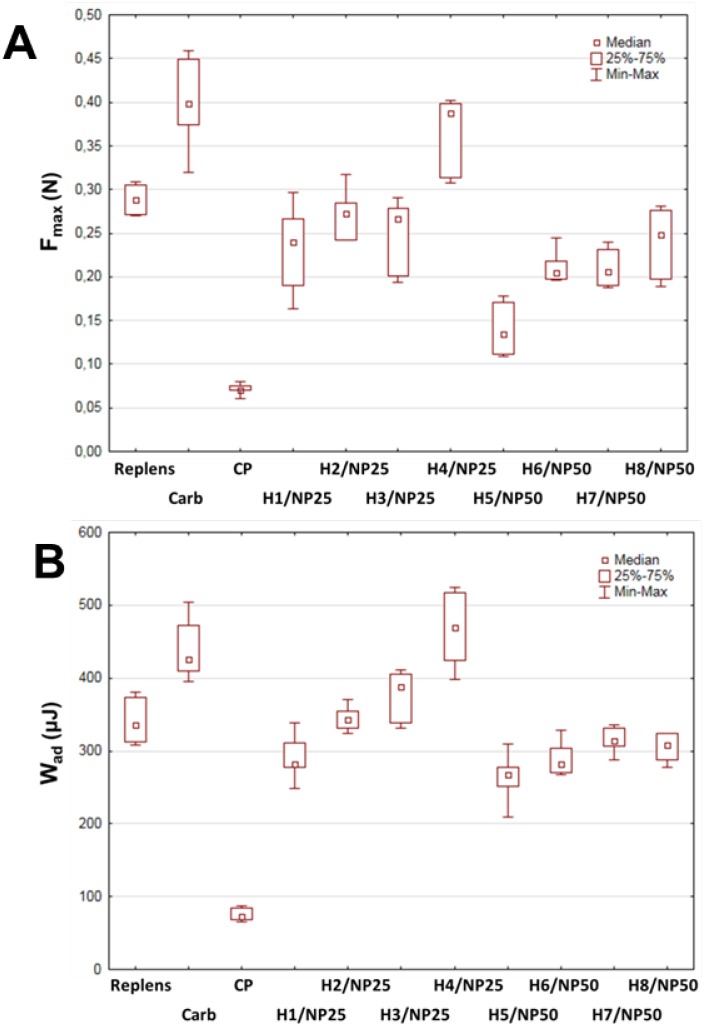
Box-plot graphs displaying mucoadhesive properties: (**A**) maximum force of detachment; and (**B**) work of adhesion of formulations H1–H4/NP25 or H5–H8/NP50 with 25 or 50 ppm TA-AgNPs concentration; commercially available vaginal gel Replens™, 0.5% (*w*/*w*) Carbopol 974P base (Carb), and cellulose paper (CP) were used as controls. Values are expressed as median (*n* = 6). Box-plot extends from the first quartile to the third quartile. Whiskers represent minimum and maximum values.

The mucoadhesive measurements of TA-AgNPs-based hydrogels to porcine vaginal mucosa were additionally accomplished using texture analyzer equipped with G/Muc measuring system. Porcine vaginal mucosa was applied for the imitation of vaginal mucoadhesion because of the similarity of porcine and human vaginas in terms of anatomical structure, vaginal secretion and pH [28]. The effect of Carbopol 974P and TA-AgNPs concentration on the force of detachment (F_max_) and work necessary to overcome the hydrogel–porcine vaginal mucosa interaction (W_ad_) is displayed in Figure 4A,B.

All examined hydrogels were found to interact with porcine vaginal mucosa, although certain variations in the F_max_ and W_ad_ parameters between formulations were noticed. As shown in Figure 4A, the strength of the mucoadhesive joints increased with the concentration of Carbopol 974P in the formulations and hydrogel H4/NP25 with 0.75% (*w*/*w*) Carbopol 974P exhibiting the highest of F_max_ and W_ad_ values (median 0.38 N and 467 μJ, respectively). In general, the hydrogels H1–H4/NP25 ability to interact with mucoadhesive material was found to be comparable with the values received for the reference gel Replens™, whereas formulations H5–H8/NP50 were characterized by slightly lower values of mucoadhesive parameters. The observed drop in mucoadhesiveness of hydrogels H5–H8/NP50 could be related to their lower viscosity and mechanical properties (Table 1), although possible interaction between silver nanoparticles and carboxylic groups cannot be excluded. In the present studies, Carbopol 974P might create complexes with TA-AgNPs which diminish the number of carboxylic groups capable of interacting with mucosal tissue and hence in consequence attenuate polymer mucoadhesive potential.

### 2.2. Assessment of In Vitro Antiviral Efficacy

Currently, elaboration of novel therapeutic approaches and effective prophylactic strategies against HSV is of great interest for public health systems worldwide. At present, anti-HSV therapy is merely limited to symptomatic treatment using nucleoside analogs (e.g., acyclovir), which act at the cellular level by impeding viral replication. Even though acyclovir and related analogs are considered first-line anti-HSV drugs with a high safety profile, viral resistance especially in immunocompromised patients is a major limitation of effective therapy [5,29]. In addition, commercially available inhibitors of viral replication do not work upon the cell-free virus released from the infected cells and therefore are not able to block further spread of the cell-free virus [30]. Therefore, particular emphasis is laid on the development of assemblies capable of preventing viral reactivation.

Among HSV-therapeutic strategies, development of topically applied microbicides is regarded as attractive approach in the prevention of HSV mucosal transmission. Several classes of microbicides are under investigation, including acid-buffering agents [31], antivirals derived from natural compounds [32] or nano-scale compounds, e.g., dendrimers [33] or heparin nanocarriers [11]. In the present studies, the potential of hydrogels loaded with TA-AgNPs as microbicide delivery system with ability to impede viral entry and interfere with its assembly was investigated.

Ideally, a microbicide agent should work by binding cell-free virus and thus blocking its further spread to neighboring uninfected cells. Simultaneously, microbicide delivery system should provide intimate contact with the mucosal tissue to prolong residence time and protect from infection by residual active virus. 

**Figure 5:**
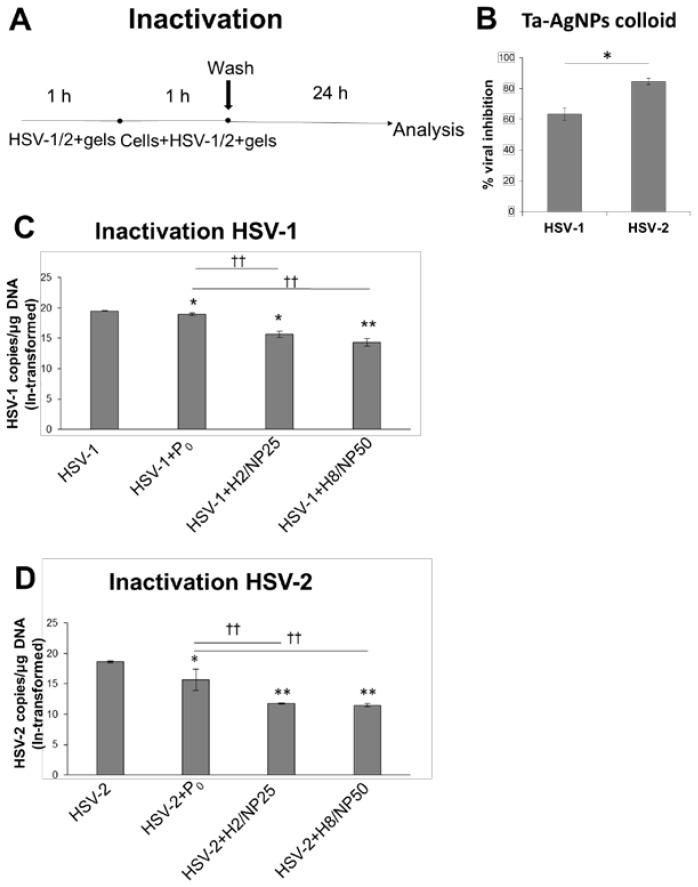
(**A**) The scheme of HSV inactivation assay. (**B**) HSV-1 or HSV-2 inhibition (%) in HaCaT cells pre-incubated for 1 h with TA-AgNPs colloid in concentration of 5 ppm. At 24 h post infection h p.i., cells were collected and titrated to determine PFU/mL in comparison to HSV-1/2 infected cultures. DNA titers (copies/μg DNA) of: (**C**) HSV-1; and (**D**) HSV-2 in HaCaT cells infected with the virus incubated with placebo hydrogel (P_0_) or TA-AgNPs-based hydrogels (H2/NP25 and H8/NP50 with 25 ppm or 50 ppm of TA-AgNPs, respectively) determined by RT-qPCR. Values are expressed as mean ± SD; * represents significant differences with *p* ≤ 0.05, while ** represents significant differences with *p* ≤ 0.01 in comparison to cells not exposed to hydrogels; †† symbolizes significant differences with *p* ≤ 0.01 in comparison to placebo.

To assess the anti-HSV activity and to elucidate the mechanism responsible for viral inhibition, TA-AgNPs-based hydrogels were tested with using four types of infection assays. Based on rheological and texture measurements, hydrogels H2/NP25 and H8/NP50 (with concentrations of 25 or 50 ppm of TA-AgNPs, respectively) with comparable values of viscosity and mechanical properties were chosen for in vitro and in vivo investigations. To exclude the impact of hydrogels components on HSV type 1 and 2 infectivity, placebo formulation (P_0_) was applied concomitantly with TA-AgNPs-based hydrogels. As a preliminary exploration of the potential of TA-AgNPs colloid against HSV, the virus inactivation assay was applied (Figure 5A). As shown in Figure 5B, nanoparticles markedly reduced infectivity of both HSV-1 and HSV-2 after incubation for 24 h (*p* ≤ 0.01) which is in agreement with previously published data [15]. The inhibition response varied markedly between HSV-1 and HSV-2 and Ta-AgNPs were found about 20% more potent against HSV-2 growth (*p* ≤ 0.01). 

To investigate whether selected formulations were capable of inactivating HSV-1 and HSV-2, placebo (P_0_) or TA-AgNPs-based hydrogels were pre-incubated with virus inocula for 1 h at 37 ± 1 °C and then the mixture of virus and hydrogel formulation was added to HaCaT cell line (Figure 5A).

The results presented in Figure 5C,D displayed that hydrogels H2/NP25 and H8/NP50 reduced HSV-1 and HSV-2 infectivity considerably (with *p* ≤ 0.05 and *p* < 0.01, respectively) as compared to control cells not exposed to hydrogels. Inactivation of HSV-2 with TA-AgNPs-based hydrogels was found to be more efficient (the estimate of HSV-2 DNA decay level was approximately 37% of control group) as compared to HSV-1 inactivation (the estimate of HSV-1 DNA decay level was approximately 25% of control group). Surprisingly, placebo formulation exhibited slight activity against HSV-2. Importantly, the rate of HSV-1 inactivation was found to significantly (*p* < 0.01) correlate with concentration of nanoparticles in hydrogels (Figure 5C) in contrast to treatment with formulations H2/NP25 and H8/NP50 which inactivated HSV-2 regardless of the nanoparticles concentration (Figure 5D). The observed findings might suggest that inactivation of both types of HSV virus was caused by direct interaction of nanoparticles with the viral envelope or its proteins, although formation of a protective hydrogel layer around the cells responsible for blocking HSV-cell interaction cannot be excluded. 

**Figure 6:**
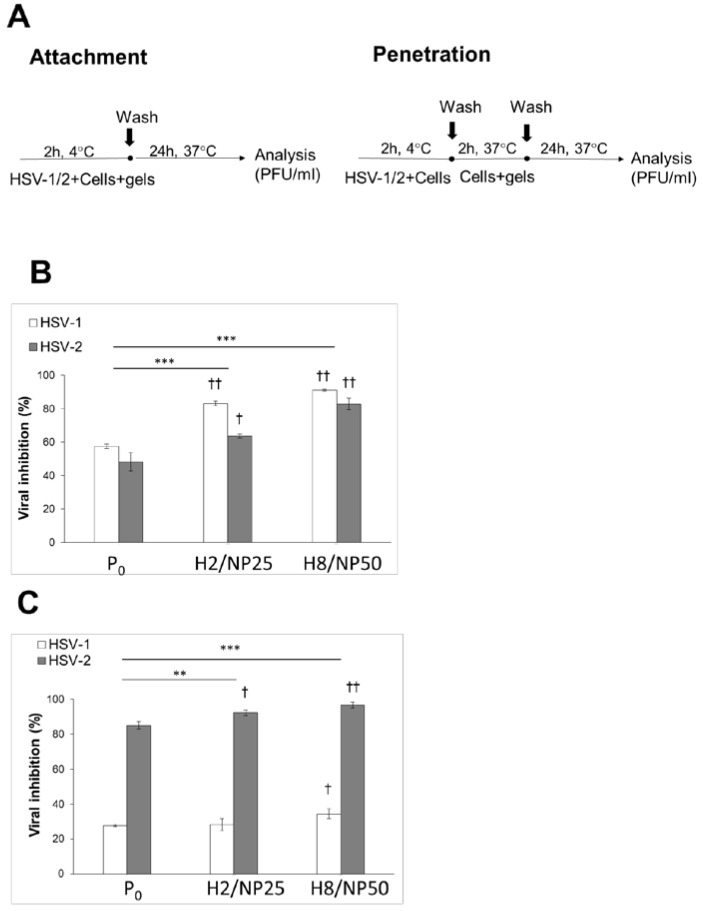
(**A**) The scheme of HSV attachment and penetration experiments; (**B**) HSV-1 or HSV-2 attachment; and (**C**) penetration inhibition (expressed in %) in HaCaT cells in the presence TA-AgNPs-based hydrogels (H2/NP25 or H8/NP50 with 25 ppm or 50 ppm of TA-AgNPs, respectively) and the corresponding placebo hydrogel (P_0_). At 24 hours post infection (h p.i.), cells and supernatants were collected and titrated to determine PFU/mL in comparison to HSV-1 or HSV-2 infected cultures. Values are expressed as mean ± SD; ** and *** represent significant differences with *p* ≤ 0.01 and *p* ≤ 0.001, respectively, in comparison to HSV-1 or HSV-2 infected untreated cultures, while † signifies substantial differences with *p* ≤ 0.05 and †† represents significant differences with *p* ≤ 0.01 in comparison to placebo.

To further examine the antiviral mode of TA-AgNPs-based hydrogels action, the viral attachment and entry assays were employed (Figure 6). To enable HSV virus binding to the cell surface, infected HaCaT cells were pre-incubated at 4 ± 1 °C, whereas, to trigger further viral penetration, the temperature was shifted to 37 ± 1 °C (Figure 6A). 

Incubation with TA-AgNPs-based hydrogels was found responsible for approximately 85–90% inhibition of HSV-1 attachment to the cell surface after 24 h p.i. and the substantial reduction in plaque numbers (above 60%) of HSV-2 was observed (Figure 6B). Formulation H8/NP50 was found more effective in preventing viral attachment, especially with regard to HSV-1. Interestingly, the rate of reduction in HSV-2 plaque size was nanoparticle concentration dependent in tested hydrogels, whereas a comparable reduction in HSV-1 plaques between H2/NP25 and H8/NP50 treated group was noted. The inhibition of HSV-1 observed in hydrogel H2/NP25 treated cell group was found higher as compared to inhibitory effect toward HSV-2 (Figure 6B).

The results of HSV penetration assay demonstrated that HSV-2 was substantially impaired for entry into HaCaT cells following exposure to hydrogels H2/NP25 and H8/NP50 (Figure 6C). When tested against HSV-1, formulation H8/NP50 displayed a decrease in plaque size (overhead 38%) after 24 h incubation, whereas hydrogel H2/NP25 was found to inhibit viral penetration in a comparable manner to placebo formulation. Importantly, both tested hydrogel formulations presented a considerably lower inhibitory effect against HSV-1 than those obtained in the attachment assay (Figure 6B).

The observed alterations in susceptibility of HSV type 1 and 2 to TA-AgNPs-based hydrogels might be related to distinct mode of HSV-1 and HSV-2 interactions with heparan sulfate proteoglycans on the surface of the host cell. Despite HSV-1 and HSV-2 display similar mechanisms of binding, penetration and subsequent cell-to-cell transmission, profound structural differences between two viruses are well described [34]. Among cell surface glycoproteins involved in the viral entry into the host cell, glycoprotein C and B are found responsible for primary interaction and subsequent attachment phase of HSV-1 and HSV-2, respectively [35,36]. The results from the attachment studies might indicate that TA-AgNPs possess higher affinity to glycoprotein C than to glycoprotein B. Superior efficiency of TA-AgNPs-based hydrogels at viral penetration stage might also depend on the proline content in HSV glycoproteins, as, according to research by Luck et al., tannic acid moieties easily interact with proline-rich proteins [37]. Particularly rich in proline residues is pro-fusion domain of HSV glycoprotein D, which was displayed crucial for triggering signaling pathway and membrane fusion at viral entry [38]. 

**Figure 7:**
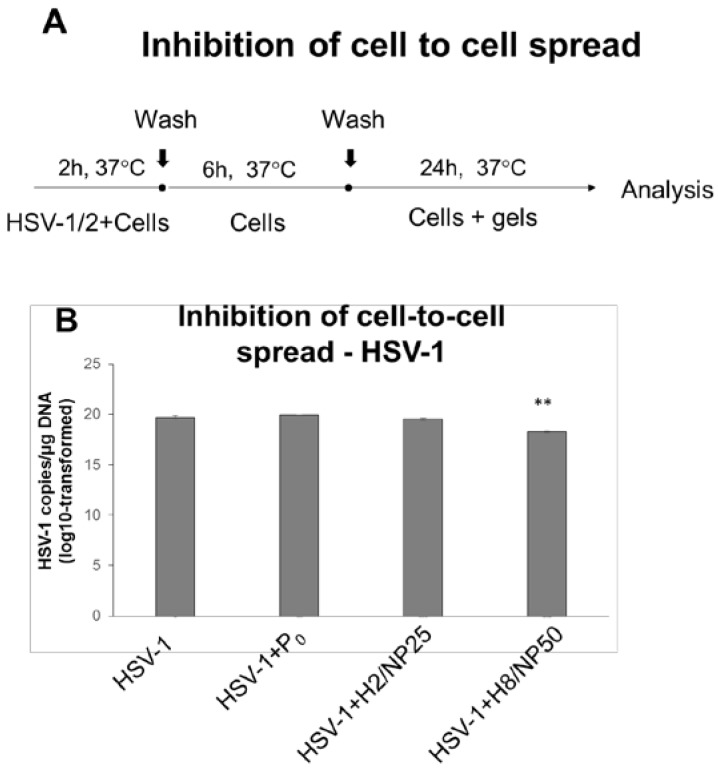

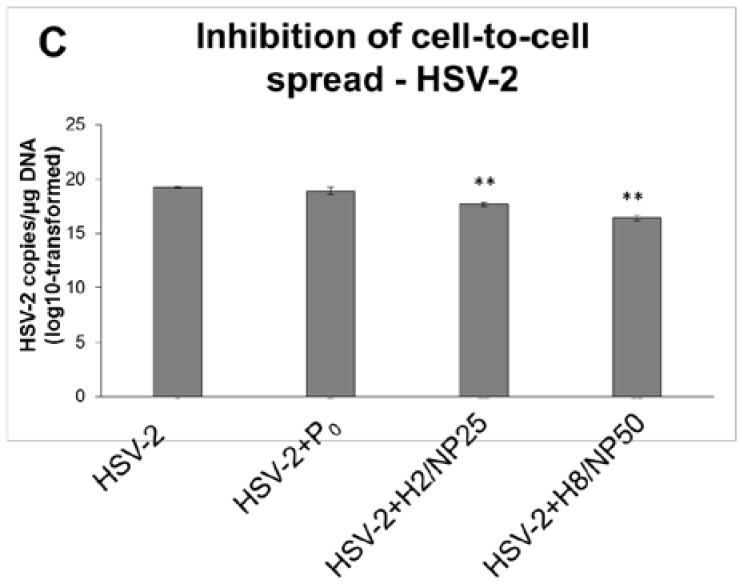
(**A**) The scheme of cell-to-cell infection assay. The results of cell-to-cell infection assay expressed as DNA titers (copies/μg DNA) of: (**B**) HSV-1; or (**C**) HSV-2 in HaCaT cells, in which TA-AgNPs-based hydrogels (H2/NP25 and H8/NP50 with 25 ppm or 50 ppm of TA-AgNPs, respectively) or the corresponding placebo hydrogel (P_0_) were added at 6 hours post infection (h p.i). Values are expressed as mean ± SD; ** represents significant differences with *p* ≤ 0.01.

Since the inhibition of HSV infectivity could be a consequence of the nanoparticles’ action inside the cell, TA-AgNPs-based hydrogels were additionally subjected to cell-to-cell spread assay in which formulations were added to HSV-1 or HSV-2 infected HaCaT cells after 8 h p.i. (Figure 7A). It was found that hydrogels H2/NP25 and H8/NP50 could prevent cell-to-cell spread of HSV-2 in infected cells markedly as compared to HSV-2 infected positive control and placebo (*p* ≤ 0.01) (Figure 7C). Interestingly, in HSV-1 infected HaCaT cell culture, exclusively hydrogel H8/NP50 with 50 ppm TA-AgNPs inhibited cell-to-cell viral transmission (*p* ≤ 0.01) (Figure 7B). 

### 2.3. In Vivo HSV Vaginal Challenge

**Figure 8:**
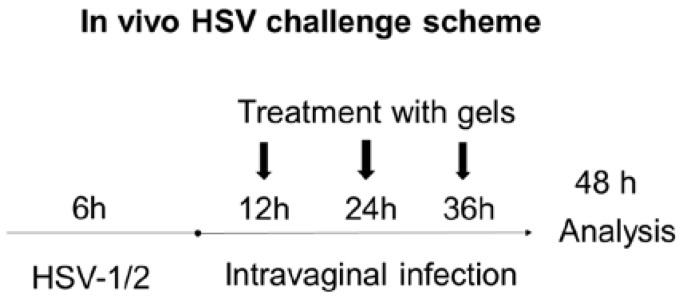
The scheme of in vivo HSV challenge with TA-AgNPs-based hydrogels or the corresponding placebo.

The experiment was carried out to demonstrate the in vivo activity of TA-AgNPs-based hydrogels for prevention vaginal HSV transmission. Formulation H2/NP25 and H8/NP50 with comparable rheological and mechanical properties enabling straightforward application to vaginal cavity were selected for the experiments. The vaginal challenge was performed with using progesterone-treated female murine vaginal model of HSV-2 genital infection. Animals were administered intravaginally with placebo or TA-AgNPs-based hydrogels every 12 h starting from 6 h p.i. according to procedure scheme presented in Figure 8. The vaginal lavages from all animals were collected at 48 h p.i. to measure the level of HSV DNA by RT-qPCR as described in Section 3.7.2.

As displayed in Table 2, post-infection treatment with TA-AgNPs-based hydrogels resulted in a marked decrease in the HSV-2 viral titers at 48 h p.i (*p* ≤ 0.05). Interestingly, treatment with formulation H2/NP25 (with TA-AgNPs concentration of 25 ppm) was found more effective with regard to HSV-2 as compared to the treatment with formulation H8/NP50 (with TA-AgNPs concentration of 50 ppm). Post-infection treatment with placebo did not result in any significant reduction in HSV replication detected in the vaginal lavages. Surprisingly, the vaginal treatment of TA-AgNPs-based hydrogels had no substantial effect on the disease scores of HSV-1 viral titers. Considering genital infections are primarily evoked by HSV type 2, the designed TA-AgNPs-based hydrogels might be considered as potential microbicide candidate to prevent transmission of genital herpes through local application. Since performed Ta-AgNPs-based hydrogels were displayed to be highly potent in vitro HSV-1 attachment inhibitors, further in vivo studies are needed to fully elaborate the potential of nanoparticle-loaded formulations against HSV-1.

## 3. Materials and Methods

### 3.1. Materials

Carbopol type 974P (Carbomer Homopolymer Type B) introduced for use in mucosal applications (with moisture content 1.7 ± 0.2%) was received as a gift sample from Lubrizol (Cleveland, OH, USA). Commercially available vaginal mucoadhesive gel Replens™ was obtained from APC Institute (Warsaw, Poland). Porcine vaginal mucosa from white pigs weighting approximately 180 kg was attained from veterinary service of local slaughterhouse (Turośń Kościelna, Poland). Freshly excised tissue was washed and frozen at −20 °C immediately after the animal died [39]. Prior the test, samples were defrosted at ambient temperature and cut into 2 cm long and 2 mm thick pieces. Silver nitrate with purity 99.99%, sodium citrate and tannic acid were purchased from Sigma Aldrich (St. Louis, MO, USA). Triethylamine (TEA) and nitric acid (HNO_3_) were obtained from Avantor Performance Materials (Gliwice, Poland).

Minimum Essential Medium with alpha modification (α-MEM), Dulbecco’s Modified Eagle Medium (D-MEM), fetal bovine serum (FBS), fetal calf serum (FCS), phosphate buffer saline (PBS), glutamine, penicillin and streptomycin were purchased from Life Sciences Technologies (Warsaw, Poland). Tissue DNA and cell culture DNA isolation kits were obtained from Eurx (Gdansk, Poland).

### 3.2. Nanoparticles

Tannic acid modified silver nanoparticles (TA-AgNPs) were obtained as defined previously by high yield and easily controllable combined reduction method (with 99.5% degree of conversion) in self-contained system [19]. Briefly, sterile aqueous solution of silver nitrate (0.017%, 95.2 g) was used as a metal salt precursor whereas sodium citrate (4%, 4.2 g) was applied as reductant agent and tannic acid (5%, 0.6 g) as reductant and stabilizing agent. TA-AgNPs colloid (with a concentration of 100 parts per million (ppm)) in water adjusted to pH 5.3 with 0.1 M HNO_3_ was characterized by nanoparticle size of 33 ± 13 nm and zeta potential of −52 ± 8 mV (examined using dynamic light scattering Nano ZS zetasizer system (Malvern Instruments, Worcestershire, UK). 

### 3.3. Cell Lines and Viruses

Green monkey kidney cell line (GMK-AH1) was a kind gift from the Swedish Institute for Infectious Disease Control (Stockholm, Sweden) and immortal human keratinocyte cell line (HaCaT)—commonly applied model to study mechanisms responsible for HSV infection and interaction of viral glycoproteins with the cell surface [40]—was a gift from Department of Clinical Virology, University of Göteborg (Göteborg, Sweden). GMK-AH1 were grown as previously described [15]. Briefly, cells were maintained in complete culture medium consisted of α-MEM with 10% FBS, 100 U/mL penicillin and 100 µg/mL streptomycin, whereas human HaCaT keratinocytes were propagated in D-MEM supplemented with 10% fetal calf serum, 10 U/mL penicillin and 100 μg/mL streptomycin [41].

HSV-1 strain McIntyre and HSV-2 strain 333 isolate were received from Department of Preclinical Sciences, Warsaw University of Life Sciences (Warsaw, Poland) and Department of Clinical Virology (Göteborg University, Göteborg, Sweden), respectively, and propagated in GMK-AH1 cells [15].

### 3.4. Preparation of TA-AgNPs-Based Hydrogels

Different hydrogel formulations with increasing Carbopol 974P concentration (0.2, 0.25, 0.3, 0.375, 0.5, 0.525 and 0.75% (*w*/*w*)) were produced to evaluate gel with proper rheological and mechanical properties for local administration. Hydrogels’ preparation process was performed under aseptic conditions in a laminar flow cabinet Lamil Plus13 equipped with HEPA filters (Karstulan Metalli Oy, Karstula, Finland). During preliminary studies, the optimized technological procedure and the order of mixing the excipients were chosen and presented as follow: the exact amount of Carbopol 974P was allowed to hydrate in ultrapure water at ambient temperature and then stirred by an automatic steering machine until homogenous dispersion appeared. The viscosity of all unneutralized dispersions was 30 ± 2 mPa·s (at 25 °C) and pH value was in the range 3.1 to 3.3. After complete hydration, polymer solution was converted into hydrogel by carefully neutralizing with TEA. Transparent gel (pH 6.6–7.5) was next autoclaved at 121 °C, 204.9 kBa for 20 min (SterilClave 24BHD, Amadar, Poland). No discoloration as well as no significant pH or viscosities variations were observed after sterilization process. Afterwards, the appropriate amount of TA-AgNPs colloid was added dropwise to the Carbopol 974P base (in weight proportion 1:3 or 1:1) with continuous stirring. Thus, 100 g of hydrogel contained 25 ppm (formulations H1–H4/NP25) or 50 ppm (H5–H8/NP50) of nanoparticles. In addition, to obtain TA-AgNPs-free formulations (placebo, P_0_) for in vitro and in vivo studies, adequate amount of ultrapure water adjusted with 0.1 M HNO_3_ to pH corresponding to TA-AgNPs colloid was added to 0.6% (*w*/*w*) Carbopol 974P base. The composition of formulations with TA-AgNPs is given in Table 1. 

### 3.5. Characerization of TA-AgNPs-Based Hydrogels

To select the optimal formulations for local administration, hydrogels with Carbopol 974P at increasing concentrations were investigated in terms of pH values, and rheological, mechanical and mucoadhesive properties.

The pH of initial Carbopol 974P bases and the final hydrogels’ formulations were estimated by a glass electrode of the pH-meter Orion 3 Star (ThermoScientific, Waltham, MA, USA) in triplicate next day after preparation.

The surface morphology of TA-AgNPs–based hydrogels was examined by transmission electron microscopy (TEM, FEI Tecnai G2 F20, FEI Company, Hillsboro, OR, USA) equipped with LaB_6_ electron source and ultrathin windowed energy dispersive X-Ray analysis system. Before TEM measurement, 10 µL of hydrogel was placed on the 400 mesh copper grid coated with carbon film, air-dried and studied at 200 kV. The particle size range was estimated based on the measurements of more than 100 particles present in at least three areas of observation (high magnification TEM image, scale bar 100 nm).

The rheological behavior of the hydrogels was assessed at 25 ± 1 °C and 37 ± 1 °C using a digital rotational Brookfield RVDV III Ultra Viscometer (Brookfield Engineering Laboratories, Middlebro, MA, USA). Hydrogels (0.5 g) were located in the thermostated sampler holder of the viscometer, allowed to equilibrate for 10 min at 25 ± 1 °C or 37 ± 1 °C, and then C-52 spindle (with the shear rate range from 2 to 20 1/s) was lowered into the sample. The shearing time was 30 s. Measurements of apparent viscosity were carried out at 10 1/s. Each experiment was performed three times.

The mechanical properties were tested using TA.XT. Plus Texture Analyser (Stable MicroSystem, Godalming, UK) equipped with a 5 kg load cell and backward extrusion measuring system A/Be. Each formulation (30.0 ± 0.2 g) was filled in a measuring container 10 min prior to the test and then the Plexiglas disc (with diameter 25 mm) was compressed with a speed of 2 mm/s into the sample to a defined depth of 10 mm at 25 ± 2 °C. The experimental parameters were selected according to Cevher et al. with modifications [26]. The subsequent measurements were carried out with 15-min intervals—the empirically set time prerequisite for hydrogels’ structure recovery. Force–time curves were attained, the values of consistency and adhesiveness were calculated and hardness parameter was recorded using Texture Exponent 32 software [42]. Each analysis was carried out five times. 

The stability of the nanoparticle-based hydrogels was evaluated by measuring viscosity, pH and TEM analysis after 14-day storage at ambient conditions. 

### 3.6. Ex Vivo Mucoadhesion Measurements

Texture Analyser TA.XT. Plus (Stable Microsystems, Godalming, UK) equipped with a cylinder probe and the measuring system A/GMP (24 mm diameter) for semi-solid formulations was applied for the mucoadhesion test. The ex vivo mucoadhesive properties of hydrogels were assessed on porcine vaginal mucosa, which was fixed by biadhesive tape to the platform below the tester probe. The experiments were conducted at 37 ± 2 °C with an acquisition rate of 200 points/s and a trigger force of 0.003 N. A sample of each hydrogel (1.5 mL) was set on the upper probe and secured with the attached support collar to uphold the sample whilst it was setting. The support collar was removed prior analysis to completely expose the hydrogel surface. Afterward, the probe was lowered onto the surface of the porcine vaginal mucosa with a constant speed of 0.5 mm/s. After keeping a contact time for 100 s under an initial contact force 0.5 N, the two surfaces were separated at a constant rate of 0.1 mm/s. The maximum detachment force F_max_ as a function of displacement was registered directly from Texture Exponent 32 software, whereas the work of adhesion (W_ad_) was calculated from the area under the force versus distance curve [43]. Commercially available mucoadhesive gel Replens™ and 0.5% (*w*/*w*) Carbopol 974P base were applied as positive controls and cellulose paper as negative control. Each experiment was performed at least six times.

### 3.7. HSV Infection In Vitro

#### 3.7.1. Virus Plaque Forming Assay

Plaque forming analysis was performed according to Navarro et al. with modifications [44] to evaluate infection levels including measurements of cytopathic effect. Briefly, cell monolayers were infected with serial dilutions of HSV-1 or HSV-2 preparations (from 10-3 to 10-6) in α-MEM with 10% FBS, 10 U/mL penicillin and 100 μg/mL streptomycin for 3 h at 37 ± 1 °C. Next, cells were washed and fresh medium supplemented with 2% low gelling temperature agarose (Sigma Aldrich, Germany) was added. After 48 h, the plates were fixed with 10% formaldehyde in PBS for 30 min, then the agar overlay was removed, and the cells were stained with 1% solution of crystal violet in 70% methanol for 30 min. The stained monolayers were then washed and plaque forming units (PFU) were calculated under the inverted microscope. 

#### 3.7.2. Virus Quantification by Real Time Polymerase Chain Reaction

Quantitative real time polymerase chain reaction (RT-qPCR) was used for determination of number of HSV-1 and HSV-2 particles present in the cells. Total DNA was purified from the vaginal lavages or HaCaT keratinocytes using Tissue DNA and Cell culture DNA isolation kits, respectively (Eurx, Gdańsk, Poland). The differentiation between HSV-1 and HSV-2 was based on the alterations between the probes at five nucleotide positions. For virus quantification, primers amplifying glycoprotein B fragment 742 bp of HSV-1 (HSV-1glyL (GTGATGTTGAGGTCGATGAAGGT) and HSV-1glyR (ACAACGCGACGCACATCAAGGT)) or fragment 1137 bp of HSV-2 (HBV2glyL (CGTACGATGAGTTTGTGTTGGCGA) and HBV2glyR (TCAGCTGGTGAGAGTACGCGTA)) were designed and used in qPCR reaction. Fragment 742 bp of HSV-1 glycoprotein B was resolved on and next extracted from 1% agarose gel. Fragment 1137 bp of HSV-2 glycoprotein B was cloned in pGEM Easy T vector (Promega, Madison, WI, USA) in *Escherichia coli* followed by plasmid isolation. The concentrations of 742 bp fragment of HSV-1 glycoprotein B and pGEM Easy T/HSV-2 glycoprotein B were assessed spectrophotometrically and expressed as number of copies per µL. The calculations were based on the molecular mass of DNA sequences: 458,629.50 g/mol and 2,565,710.92 g/mol for 742 bp fragment of HSV-1 and pGEM Easy T/HSV-2 glycoprotein B, respectively. Real time quantitative polymerase chain reaction (qPCR) was performed in ABI Prism 7000 (Applied Biosystems, Carlsbad, CA, USA) and quantified as described by Namvar et al. [45]. Briefly, all PCR reactions were performed in a volume of 20 µL using the following thermal profile: 50 °C (2 min), 95 °C (10 min), 40 cycles: 95 °C (15 s) and 58 °C (1 min). PCR reactions consisted of 1 × TaqMan™ Universal PCR Master Mix (Thermo Fisher Scientific, Waltham, MA, USA), 0.9 μM HSV1-F primer (GCAGTTTACGTACAACCACATACAGC), 0.9 μM HSV1&2-R primer (AGCTTGCGGGCCTCGTT) and 0.2 μM HSV1-probe (FAM-CGGCCCAACATATCGTTGACATGGC-TMARA) to amplify 117 bp long DNA fragment of HSV-1 glycoprotein B or 0.9 μM HSV2-F primer (TGCAGTTTACGTATAACCACATACAGC), 0.9 μM HSV1&2-R primer and 0.2 μM HSV2-probe (FAM-CGCCCCAGCATGTCGTTCACGT-TAMRA) to amplify 118 bp long DNA fragment of HSV-2 glycoprotein B. Standard calibration curve was linear over the range of 12–1.2 × 107 and 11–1.1 × 107 copies per reaction for HSV-1 and HSV-2, respectively. The amount of genomic DNA sample tested for virus presence ranged 7.5–96 ng per reaction.

#### 3.7.3. HSV Inactivation Assay

Formulations H2/NP25 or H8/NP50 (with nanoparticles’ concentration of 25 or 50 ppm, respectively) with comparable values of viscosity and mechanical properties were selected for HSV inhibition studies. TA-AgNPs colloid, TA-AgNPs-free hydrogel (placebo, P_0_) or TA-AgNPs-based hydrogels ten-fold diluted in complete culture medium (D-MEM, 10% FCS, 10 U/mL penicillin and 100 μg/mL streptomycin) were incubated with HSV-1 or HSV-2 inocula (with a multiplicity of infection (MOI 5)) for 1 h at 37 ± 1 °C. Next, mixture of virus and proper formulation was added to HaCaT cells seeded in 24-well plates (1 × 105 cells per well). The control comprising HSV not exposed to the hydrogel formulations. After 1 h post infection (h p.i.) cells were washed twice with ice cold PBS to remove unattached virus and hydrogel formulations, overlaid with 1 mL of fresh complete culture medium and incubated at 37 ± 1 °C (Figure 5A). At 24 h p.i., cells were collected and analyzed by RT-qPCR to determine HSV-1 or HSV-2 DNA copy number per µg DNA.

#### 3.7.4. HSV Attachment Assay

The effect of hydrogel formulations on viral attachment was evaluated according to method described previously [15]. Concisely, TA-AgNPs-free hydrogel (placebo, P_0_) or TA-AgNPs-based hydrogels (H2/NP25 and H8/NP50) ten-fold diluted in complete culture medium were added to 24-well plates containing pre-chilled (at 4 ± 1 °C for 1 h) HaCaT cell monolayers (1 × 105 cells per well) infected with HSV-1 or HSV-2 (MOI 1). To allow viral attachment, plates were incubated for 2 h at 4 ± 1 °C (Figure 6A). To remove unattached hydrogels and unabsorbed HSV, inocula were removed, HaCaT cells were rinsed twice with ice cold PBS and overlaid with fresh culture medium, followed with further incubation at 37 ± 1 °C. At 24 h p.i., the infected cells were analyzed by plaque forming assay and percent inhibition of viral attachment was calculated by setting the number of plaques attained in control.

#### 3.7.5. HSV Penetration Assay

HSV-1 or HSV-2 inocula (MOI 1) were placed in 24-well plates containing pre-chilled (at 4 ± 1 °C for 1 h) HaCaT cell monolayers (1 × 105 cells per well) and incubated for 2 h at 4 ± 1 °C to allow virus binding [46]. Unattached inocula were then removed and HaCaT cells were washed twice with ice cold PBS. Next, TA-AgNPs-free hydrogel (placebo, P_0_) or TA-AgNPs-based hydrogels (H2/NP25 and H8/NP50) ten-fold diluted in complete culture medium were added to the plates and incubated for 2 h at 37 ± 1 °C to trigger viral penetration. Afterwards, the formulations were removed, cells were washed twice with ice cold PBS and overlaid with 1 mL culture medium. After 18 h of incubation at 37 ± 1 °C, HaCaT cell culture was subjected to the plaque forming assay (Figure 6A). Percent inhibition of viral penetration was calculated by setting the number of plaques attained in control.

#### 3.7.6. HSV Cell-to-Cell Infection Assay

For examining post viral entry effects of hydrogels (inhibition of cell-to-cell spread), HaCaT keratinocytes were infected at 37 ± 1 °C with HSV-1 or HSV-2 (MOI 1) (Figure 7A). Following the adsorption and penetration period (2 h at 37 ± 1 °C), the inocula were removed, HaCaT cells were subsequently washed twice with ice cold PBS and overlaid with fresh culture medium. At 6 h p.i., the time prerequisite to transfer the virus to uninfected cells, TA-AgNPs-free hydrogel (placebo, P_0_) or TA-AgNPs-based hydrogels (H2/NP25 or H8/NP50) ten-fold diluted in complete culture medium were added to inoculum. After incubation for 24 h at 37 ± 1 °C the infected cultures were further analyzed by qPCR to determine HSV-1 or HSV-2 copies/μg DNA.

### 3.8. In Vivo HSV Challenge

Female C57BL/6 mice, which were 6–8 weeks old, obtained from Mossakowski Medical Research Centre (Warsaw, Poland), were used for all experiments. The experimental protocol was approved by the Local Committee on the Ethics of Animal Experiments in Warsaw, Poland, and performed in strict accordance with the recommendations of Directive 2010/63/EU of the European Parliament and the Council of 22 September 2010 on the protection of animals used for scientific purposes. Preceding to vaginal HSV-1 or HSV-2 infection, mice received a subcutaneous injection of medroxyprogesterone acetate in a dose 2.0 mg/kg body weight (b.w.) in 100 µL of PBS (Depo-Provera; Upjohn Puurs-Belgium Pfizer, Germany) in orde to increase their susceptibility to viral infection. Five days after injection, mice were anaesthetized with an intraperitoneal injection (0.1 mL/10 g b.w.) of ketamine (9 mg/mL) and xylazine (1 mg/mL) and inoculated intravaginally with HSV-1 or HSV-2. A viral dose of 104 PFU in 20 µL PBS was used for all infections. After administration animals were maintained in supine position for 15–30 min. After 6 h p.i., mice were randomized into four groups (5 mice per group): control group, with no treatment; placebo group, administrated with 35 µL of hydrogel P_0_; and two groups that received vaginally 35 µL of TA-AgNPs-based hydrogels (H2/NP25 or H8/NP50). Each hydrogel formulation was administered with using micropipette. Application of gels was repeated 3 times every 12 h. At 48 h following infection, mice were sacrificed and the vaginal lavages were performed for virus quantification by RT-qPCR. 

### 3.9. Statistical Analysis

Quantitative variables were expressed as the mean ± standard deviation (SD) or the median. Statistical analysis was accomplished using nonparametric Kruskal–Wallis test for mucoadhesive assessment, and, for HSV infection in vitro and in vivo, Wilcoxon test was used (Statistica 12.5 software, StatSoft, Kraków, Poland). Differences between groups were reflected to be significant at *p* < 0.05. For in vitro studies, at least three independent experiments were carried out with duplicate or triplicate wells for control. The number of mice in each group was estimated with the power level set at 0.8 and the conventional level alpha 0.05 for a paired *t*-test.

## 4. Conclusions

This work demonstrates the feasible approach to formulate multifunctional mucoadhesive hydrogel by incorporating TA-AgNPs into Carbopol 974P gel after crosslinking with gelation initiator. In the rheological and mucoadhesive measurements, thixotropic properties of obtained preparations and their profound ability to interact with mucosal membrane in the ex vivo porcine vaginal model were demonstrated. The results presented in this study indicate that hydrogels with TA-AgNPs possess distinctive ability to prevent both HSV-1 and HSV-2 infection by direct inhibition of viral attachment, penetration and post-infection spread. Notably, substantial differences in activity of TA-AgNPs-based hydrogels toward HSV-1 and HSV-2 were demonstrated, suggesting distinct mode of anti-HSV action according to the virus type and/or type of mucosal tissue. The nanoparticle-based hydrogels’ impact at viral attachment stage appeared to play a substantial role in HSV-1 inhibition, whereas activity against HSV-2 was mediated by blocking attachment as well as penetration stage. Interestingly, profound anti-HSV-2 effect by corresponding placebo hydrogel was observed during the penetration assay. suggesting additional impact of the mucoadhesive semi-solid dosage form in HSV-therapy. However, these hypotheses need further investigation to explore the influence of drug carrier as potent antiviral adjunctive to Ta-AgNPs.

The findings from the HSV infectivity studies added novel insight into the potential efficacy of TA-AgNPs-based hydrogels in local treatment of HSV infections, although additional in vivo studies are needed to provide more specific data concerning the role of nanoparticles in prevention strategy of HSV infections.

## Figures and Tables

**Table 1:** Composition of TA-AgNPs-based hydrogels and their physicochemical and mechanical properties; results are expressed as mean ± standard deviation (SD).

Component (g)			Formulation							
	Control	P_0_	With TA-AgNPs 25 ppm a				With TA-AgNPs 50 ppm b			
			H1/NP25	H2/NP25	H3/NP25	H4/NP25	H5/NP50	H6/NP50	H7/NP50	H8/NP50
TA-AgNPs colloid		-	25.0	25.0	25.0	25.0	50.0	50.0	50.0	50.0
Carbopol 974P	0.5	0.60	0.40	0.50	0.75	1.0	0.40	0.50	0.75	1.0
TEA	1.0	0.9	0.6	1.0	1.12	1.5	0.6	1.0	1.12	1.5
Parameter										
pH (*n* = 3)	7.3 ± 0.3	7.1 ± 0.1	6.6 ± 0.1	6.6 ± 0.2	7.4 ± 0.2	7.5 ± 0.1	6.6 ± 0.2	7.0 ± 0.3	6.9 ± 0.1	7.0 ± 0.1
Viscosity at 25 ± 1 °C (mPa·s) (*n* = 3) c	n.d.	7520 ± 131	4921 ± 115	7005 ± 102	7000 ± 124	14970 ± 198	3910 ± 98	3940 ± 101	6610 ± 108	7670 ± 134
Viscosity at 37 ± 1 °C (mPa·s) (*n* = 3) c	n.d.	5080 ± 152	3950 ± 78	4480 ± 89	6290 ± 106	12915 ± 198	3490 ± 102	3670 ± 84	4500 ± 85	5530 ± 112
Hardness (g) (*n* = 5)	248.1 ± 21.0	206.4 ± 29.0	178.4 ± 20.1	207.3 ± 4.6	267.1 ± 18.1	542.6 ± 29.9	155.6 ± 9.1	167.2 ± 11.9	191.4 ± 7.9	218.3 ± 10.1
Adhesiveness (g·s) (*n* = 5)	3498 ± 109	3210 ± 125	2820 ± 186	3041 ± 79	3210 ± 250	3894 ± 198	1633 ± 156	1710 ± 148	1900 ± 109	2851 ± 185
Consistency (g) (*n* = 5)	441.8 ± 9.8	352.8 ± 39.6	341.4 ± 19.2	391.9 ± 15.3	471.6 ± 28.0	801.7 ± 27.8	251.2 ± 19.1	302.5 ± 14.0	348.2 ± 20.1	378.4 ± 22.7

Silver nanoparticles modified with tannic acid (TA-AgNPs) colloid to Carbopol base ratio (*w*/*w*) was a 1:3 or b 1:1; c defined in half the maximum shear rate value; n.d., not determined.

**Table 2:** HSV-1 and HSV-2 DNA loads (expressed as copies/μg DNA, ln-transformed) in the vaginal lavages determined by RT-qPCR at 48 hours post infection (h p.i.); data are presented as mean log-transformed data (ln) ± SD; * represents significant differences with *p* ≤ 0.05.

Virus	Control ^a^	P_0_^b^	H2/NP25 ^c^	H8/NP50 ^d^
HSV-1	15.61 ± 0.17	15.33 ± 0.24	14.42 ± 0.08	14.98 ± 0.15
HSV-2	16.58 ± 0.06	16.71 ± 0.06	12.65 ± 1.15 *	14.21 ± 0.65 *

^a^ Infected, untreated mice; ^b^ placebo hydrogel; ^c^ TA-AgNPs-based hydrogel with 25 ppm or ^d^ 50 ppm of TA-AgNPs, respectively.

## Abbreviations

b.w.	Body weight
Carb	Carbopol type 974P (Carbomer Homopolymer Type B)
D-MEM	Dulbecco’s modified eagle medium
FBS	Fetal bovine serum
FCS	Fetal calf serum
F_max_	Force of detachment
GMK-AH1	Green monkey kidney cell line
GRAS	Generally regarded as safe
HaCaT	Immortal human keratinocyte cell line
HSV	Herpes simplex virus
h p.i.	Hours post infection
α-MEM	Minimum essential medium with alpha modification
MOI	Multiplicity of infection
P_0_	Nanoparticle-free hydrogel (placebo)
ppm	Parts per million
PBS	Phosphate buffer saline
PFU	Plaque forming units
SD	Standard deviation
RT-qPCR	Real time quantitative polymerase chain reaction
TA-AgNPs	Tannic acid modified silver nanoparticles
TEA	Triethylamine
TEM	Transmission electron microscopy
*w*/*w*	Weight/weight
W_ad_	Work of adhesion
